# First Confirmed Detection of *Gnathotrichus materiarius* (Fitch, 1858) (Coleoptera: Curculionidae: Scolytinae) in Slovakia

**DOI:** 10.3390/insects17050532

**Published:** 2026-05-21

**Authors:** Michal Lalík, Juraj Galko, Christo Nikolov, Andrej Kunca, Slavomír Rell, Milan Zúbrik, Jozef Vakula, Andrej Gubka, Roman Leontovyč, Jaroslav Holuša

**Affiliations:** 1 National Forest Centre, Science and Research Section, T. G. Masaryka 2175/22, 960 01 Zvolen, Slovakia; 2 Faculty of Forestry and Wood Sciences, Czech University of Life Sciences, Kamýcká 129, 16501 Prague, Czech Republic

**Keywords:** ambrosia beetle, invasive species, first record, pine stands, pheromone traps, monitoring

## Abstract

*Gnathotrichus materiarius* Fitch, 1858 is a North American ambrosia beetle that has progressively expanded its distribution across Europe during the twentieth and twenty-first centuries. The aim of this study was to assess its occurrence in Slovakia based on multi-year surveys conducted in the Záhorie region and to document its first national record. Monitoring was carried out in 2021 and 2025 in pine-dominated stands using traps baited with pheromone and kairomone attractants. No individuals were detected in 2021. In 2025, two adults of *G. materiarius* were captured at a single site during the first inspection of traps baited with a (+)-α-pinene and ethanol blend. A large volume of stored pine timber was present in close proximity to the capture site, providing suitable breeding material. The findings suggest a recent introduction or early stage of establishment in Slovakia. Although the species is regarded as a technical pest of coniferous wood, it does not colonize healthy trees and currently poses a limited risk to vital forest stands. Continued targeted monitoring is important to assess its further spread.

## Simple Summary

*Gnathotrichus materiarius*, a North American ambrosia beetle, was recorded for the first time in Slovakia in 2025 in Scots pine stands of the Záhorie region. The species was detected exclusively at a site with accumulated pine timber, highlighting the risk of introduction through wood handling and the importance of targeted monitoring in pine-dominated forests.

## 1. Introduction

*Gnathotrichus materiarius* Fitch, 1858 is a North American ambrosia beetle native to the Nearctic region [1]. The species was first detected in Europe in France in 1933 [2] and has since progressively expanded its distribution across the continent, including Germany 1964 [3], Netherlands 1965 [4], Belgium 1979 [5], Switzerland 1984 [6], Sweden 1986 [7], Italy 1993 [8], Finland 1996 [9], Spain 2003 [10], Czech Republic 2005 [11], Slovenia 2007 [12], Austria 2012 [13], United Kingdom 2013 [14], Poland 2015 [15] and Hungary 2016 [16].

Adults are less than 3.5 mm long, nearly cylindrical, and more than three times as long as they are wide. The dorsum is smooth, slightly shining, and dark reddish–brown to blackish–brown. The head is deflexed, blackish, and moderately punctate, with a slightly elevated smooth median line. The antennal club is about 1.5 times as long as the funicle. Females bear several long, curved setae on the outer side of the club and funicle, which are absent in males. The pronotum is not constricted before the middle, with an elevated transverse carina. Each fore tibia has three blunt teeth at the apex. The elytra are about twice as long as wide, with parallel sides, and a narrowly rounded posterior margin. The strial punctures are fine and arranged in regular rows [17]. The species is monogamous, with an approximately 1:1 sex ratio and no evidence of sibling mating or parthenogenesis [18,19]. Both larvae and adults feed on the symbiotic fungus *Ambrosiozyma monospora* (Saito) (syn. *Endomycopsis fasciculata*) [20,21,22,23], which has been recorded in both the native and introduced ranges of the species [20,24]. Adults are active throughout the vegetation period, with peak flight typically occurring in May or June [23,25].

*G. materiarius* is regarded as a secondary pest of coniferous timber in North America and Europe [25,26,27]. The species shows a preference for *Pinus* spp., but has been recorded from numerous conifer genera, including *Abies* Mill., *Larix* Mill., *Picea* A.Dietr., *Pinus* L., *Pseudotsuga* Carrière, *Thuja* L., and *Tsuga* Carrière [17,18,28]. It colonizes dead and declining trees, and the damage caused to date has been limited to gallery formation and wood discoloration associated with its symbiotic fungus [9,18]. The species has also been reported to colonize debarked timber [29].

No species-specific aggregation pheromone has been identified for *G. materiarius*; however, adults can be attracted to host material [30] and are frequently captured in traps baited for *Ips* spp. [31,32,33]. Commonly used attractants for this purpose include ID Ecolure^®^, IT Ecolure^®^, Cembräwit^®^, Amitinuswit^®^, Hostowit^®^, and ethanol in combination with other lures [11,18,30,34].

The spread of ambrosia beetles in Europe is frequently associated with the transport and storage of coniferous timber, wood packaging material, and other wood products. Early detection of newly introduced species is therefore essential for assessing establishment risk and preventing further spread within managed forest ecosystems.

The objective of this study was to evaluate the occurrence of *G. materiarius* in western Slovakia based on trapping surveys conducted in two separate years and to document its first confirmed record in the country in 2025. The Slovak name for this species is drvinárik podlhovastý.

## 2. Materials and Methods

### 2.1. Study Area and Sampling Design

**Figure 1:**
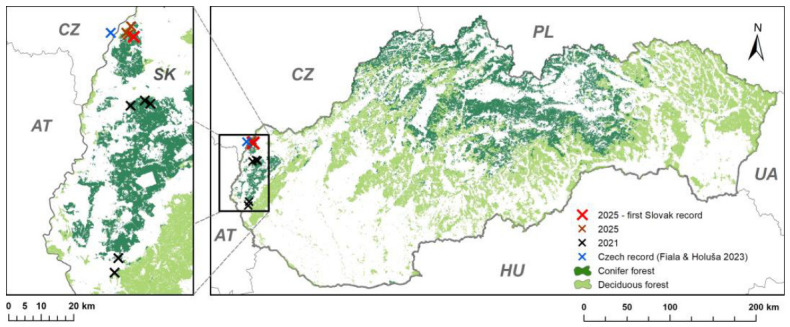
Location of monitoring sites in the Záhorie region (Slovakia) where traps were installed in 2021 and 2025. The first Slovak record of *Gnathotrichus materiarius* (2025) was obtained at 48.742544° N, 17.084601° E. The map also shows the nearest previously reported occurrence of the species in the Czech Republic [34]. Country abbreviations indicate the following countries: CZ—Czech Republic, AT—Austria, PL—Poland, HU—Hungary, and UA—Ukraine.

Monitoring of the target species was conducted during two sampling periods, in 2021 and 2025, in the Záhorie region (western Slovakia) (Figure 1) in pine-dominated forest stands representing suitable habitats for the species. In 2021, a total of ten traps were installed at five sites, with two traps per site. Lindgren funnel traps (Synergy Semiochemicals Corp. 7572 Progress Way, Delta, British Columbia, V4G 1E9 Canada) were used. The geographic coordinates (WGS84) of the sites were as follows: 48.621478° N, 17.130777° E; 48.627154° N, 17.115244° E; 48.617949° N, 17.075829° E; 48.313234° N, 17.031879° E and 48.339865° N, 17.042213° E. All stands consisted of 100% Scots pine (*Pinus sylvestris* L.) (Figure 1). The experiment was established on 26 May 2021; traps were inspected twice during the monitoring period, and sampling was terminated on 7 July 2021. The second monitoring was conducted in 2025 at four sites in the Záhorie region with the following coordinates: 48.742544° N, 17.084601° E; 48.762184° N, 17.077547° E; 48.750762° N, 17.064220° E, and 48.746435° N, 17.068960° E (Figure 1).

Stand composition varied among sites. Most sites consisted of 100% Scots pine, instead of the third one, that comprised a mixed stand of Scots pine (*Pinus sylvestris* L., representation of 50%) and black pine (*P. nigra* Arnold, 50%). Monitoring in 2025 was initiated on 5 May 2025 and concluded on 28 July 2025.

### 2.2. Traps and Attractants

In 2021, Lindgren funnel traps were used and baited with commercial pheromone dispensers IT Ecolure and ID Ecolure (Fytofarm s.r.o., Bratislava, Slovakia), which are known to attract ambrosia and bark beetles. At each site, one trap was equipped with IT Ecolure and the other with ID Ecolure, ensuring equal representation of both lure types across all sites.

In 2025, New 5-Unit Multi-Funnel traps (Synergy Semiochemicals Corp., Delta, BC, Canada) were used. The attractant consisted of (+)-α-pinene (98% purity) (Merck Life Science, Riedstraße 2, 89555 Steinheim, Germany) diluted in 70% ethanol (Sklochem–Agroekolab, Zvolen, Slovakia) at a ratio of 1:3 (α-pinene:ethanol). A total volume of 50 mL of the mixture was applied per trap. The attractant was placed in a 100 mL plastic bottle perforated with 100 holes (3 mm in diameter) to allow gradual and continuous release of volatile compounds throughout the exposure period.

### 2.3. Trap Installation and Sample Processing

Traps were installed within forest stands to minimize mutual interference and were placed under comparable microhabitat conditions to ensure similar exposure to environmental factors. In 2025, traps were inspected at three-week intervals throughout the monitoring period. During each inspection, captured specimens were collected, and the attractant was replenished as necessary. All collected material was transported to the laboratory, where specimens were sorted and identified using standard entomological procedures using published identification keys [17].

Despite minor mechanical damage (loss of some appendages), the specimens retained the principal diagnostic features, including body shape, antennal structure, pronotal carina, and elytral sculpture, which enabled reliable identification.

### 2.4. Photography and Image Processing

Photographs of the specimens were taken using a Leica M205 FCA stereomicroscope (Leica Microsystems GmbH, Wetzlar, Germany) with an integrated digital imaging system. Image stacks from multiple focal planes were combined to obtain extended depth-of-field photographs. Final images were slightly optimized (brightness, contrast, and sharpness) using Adobe Lightroom 6.

## 3. Results

**Figure 2:**
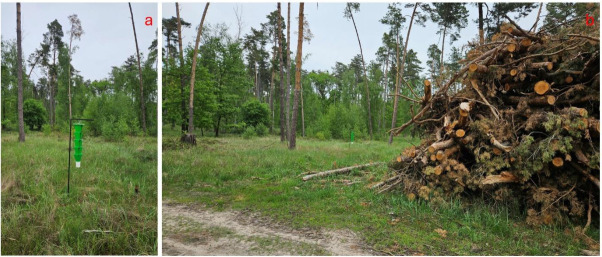
Trap used for capturing *Gnathotrichus materiarius* (**a**); locality of the first record in the Záhorie region Slovakia (**b**).

No individuals of *G. materiarius* were captured during the 2021 monitoring period. In 2025, two adults of *G. materiarius* were captured during the first trap inspection at site (48.742544° N, 17.084601° E) (Figure 2). Identification was carried out based on morphological characteristics using a determination key [35], and these characters unequivocally confirmed that the specimens belong to the species *G. materiarius.* The specimens were collected from a trap baited with (+)-α-pinene and ethanol. No additional individuals were recorded at the remaining three sites.

**Figure 3:**
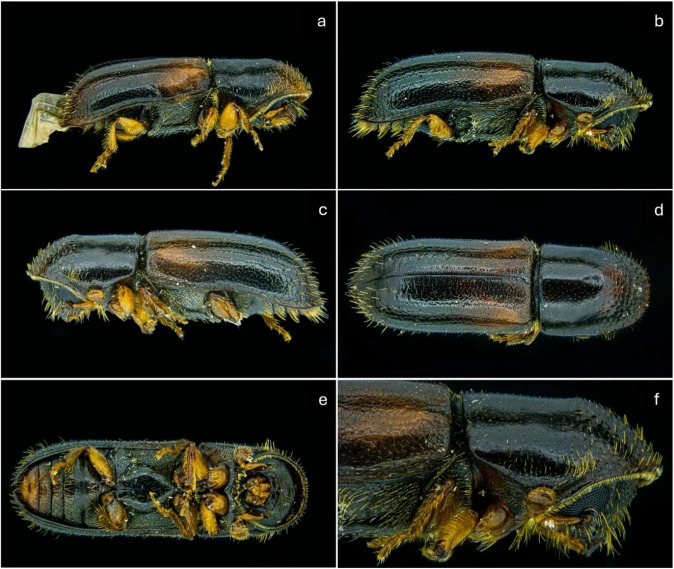
Two male adults of *Gnathotrichus materiarius* (**a**,**b**); left lateral view (**c**); dorsal view (**d**); ventral view (**e**); detail of the head (**f**). The specimens show minor mechanical damage, while key diagnostic characters remain intact.

A large volume of stored pine timber intended for wood chipping was present in the immediate vicinity of the positive trapping site (Figure 3).

## 4. Discussion

The first record of *G. materiarius* in Slovakia further extends its known secondary range in Europe. Since its initial detection in France in 1933, the species has gradually spread to many European countries, including several in Central Europe [34]. The finding in Slovakia therefore follows the broader pattern of its ongoing expansion across the continent. The Slovak record lies only a few kilometers from the nearest previously reported occurrence in the Czech Republic, suggesting that the species may already be established in the transboundary region.

Adults were captured at a single site where accumulated pine timber was present, which is consistent with the ecological requirements of the species. *G. materiarius* colonizes dead or declining coniferous wood and has also been reported from debarked timber [9,18,29]. The presence of freshly stored pine material intended for wood chipping likely provided suitable conditions for colonization and reproduction.

The absence of *G. materiarius* during the 2021 monitoring period suggests that the species was either not yet present in Slovakia at that time or occurred at very low population densities. A similar pattern has been reported in other European countries, where initial detections were sporadic and population numbers increased only in the years following introduction [9,18].

In 2025, only two individuals were captured at a single site, indicating low abundance consistent with an early stage of colonization. Such low capture numbers are typical for recently introduced ambrosia beetles before local populations become more widely established. *G. materiarius* is a monogamous species with a 1:1 sex ratio and no evidence of sibling mating or parthenogenesis, traits that may slow population growth during the initial phase of invasion [18,19].

The capture site was located in close proximity to a large volume of pine timber intended for wood chipping. This spatial association suggests a possible link between the occurrence of *G. materiarius* and the availability of freshly stored coniferous material suitable for colonization. Ambrosia beetles are commonly associated with timber transport and storage, and such locations may act as points of introduction or temporary establishment [14]. Although species-specific monitoring protocols for *Gnathotrichus materiarius* are not yet well established, our results demonstrate that general trapping approaches based on ethanol and host-derived volatiles, such as (+)-α-pinene, can be effectively used for its detection, even at low population densities [34]. Such semiochemical-based methods are widely applicable and can be readily implemented in surveillance programs across different regions and forest types. In particular, monitoring efforts should prioritize high-risk sites associated with timber storage, processing, and transport, as these environments provide suitable breeding material and represent key pathways for the introduction and spread of ambrosia beetles. The exclusive detection of *G. materiarius* at a site with accumulated pine timber in this study supports the importance of such targeted surveillance strategies for the early detection of newly introduced species.

The use of a (+)-α-pinene and ethanol blend proved effective for detecting the species. Although *G. materiarius* does not possess a species-specific aggregation pheromone, it is frequently captured in traps baited for *Ips* spp., including IT Ecolure, ID Ecolure, and ethanol in combination with other attractants [18,30,31,32,33,34]. Our results support the importance of host-derived kairomones in the monitoring of ambrosia beetles and confirm that general bark beetle lures can facilitate early detection of non-native xylomycetophagous species.

From an economic perspective, *G. materiarius* is regarded as a technical pest of coniferous timber, with damage limited to gallery formation and wood discoloration caused by the symbiotic fungus *Ambrosiozyma monospora* (Saito) van der Walt [9,18,20,21,22,24]. As the species does not colonize healthy trees, its current impact on forest ecosystems in Slovakia appears limited.

Nevertheless, further spread can be expected in relation to the availability of suitable breeding substrates and the handling of coniferous timber, as has been observed in other parts of Europe [18,24,27]. For this reason, continued targeted monitoring of the species in coniferous stands and places with increased amounts of stored or processed timber is justified.

## Data Availability

The original contributions presented in this study are included in the article. Further inquiries can be directed to the corresponding author.
